# The Impact of COVID-19 Outbreak (2nd Wave) on Mental Health of the Healthcare Community in the NHS: A Web-Based Questionnaire Study

**DOI:** 10.1192/bjo.2022.89

**Published:** 2022-06-20

**Authors:** Omer Nasim, Muhammad Khizar Hayat, Zeinab Hussain, Malghalara Afridi, Raza Ali Khan

**Affiliations:** 1 Poole General Hospital, Poole, United Kingdom; 2 Rehman Medical Institute, Peshawar, Pakistan; 3 Stockport NHS Foundation Trust, Manchester, United Kingdom

## Abstract

**Aims:**

To determine the mental impact the second wave of COVID-19 has had on health care professionals working in the National Health Services (NHS), United Kingdom.

**Methods:**

A cross-sectional descriptive web-based survey was conducted among the staff of National Health Services (NHS) in Poole, United Kingdom. Two tertiary care hospitals staff were part of this study. The study was spanned over a duration of 6 months, October 2020 to April 2021. A standard GAD-7 and PHQ-9 questionnaire along with demographic information was uploaded on google docs for data collection. All healthcare staff working in the hospitals were included. Any person that did not fill the questionnaire completely was excluded. Data collected were analysed using SPSS for descriptive statistics and the chi-squared test was done keeping p < 0.05 as significant.

**Results:**

A total of 160 health care professionals took part in the survey, with a mean age of 37.36 (SD = 11.51) years, predominantly females (58.8%). The majority of participants were not depressed (78.1%, p = 0.004) nor were they anxious (85%, p = 0.008). A significant difference (p = 0.050) was seen in participant's anxiousness regarding the source of information. All other demographic parameters were not significant for differences in depression or anxiety (p > 0.05). 33.6% of the respondents agreed and 9.6% totally agreed to being terrified of contracting the coronavirus. 40.4% disagreed while 16% did not have an opinion. A similar trend was seen for the other statements. More than half (56.3% and 56.9%) of the participants answered in the affirmative that they were worried about contracting the disease and getting their living place contaminated, a staggering 91.3% were anxious about affecting their families.

**Conclusion:**

The second wave of COVID-19 has had minimal effect on the mental health of health care workers in the NHS.

